# Supplementary material for: Amorphous Solid Dispersion of Hesperidin with Polymer Excipients for Enhanced Apparent Solubility as a More Effective Approach to the Treatment of Civilization Diseases
Source: Int J Mol Sci. 2022 Dec 2;23(23):15198. doi: 10.3390/ijms232315198 (PMC9740072; doi:10.3390/ijms232315198)
Supplement: Supplementary file 1 [file ijms-23-15198-s001.zip › ijms-2034603-supplementary.pdf]

`

Article

Amorphous Solid Dispersion of Hesperidin with Polymer Excipients for Enhanced Apparent Solubility as a More Effective Approach to the Treatment of Civilization Diseases

Natalia Rosiak [1], Kamil Wdowiak [1], Ewa Tykarska [2] and Judyta Cielecka-Piontek [1,*]

[1] Department of Pharmacognosy, Faculty of Pharmacy, Poznan University of Medical Sciences,
3 Rokietnicka St., 60-806 Poznan, Poland; nrosiak@ump.edu.pl (N.R.); 76739@student.ump.edu.pl (K.W.)

[2] Department of Chemical Technology of Drugs, Poznan University of Medical Sciences, 6 Grunwaldzka St., 60-780 Poznan, Poland; etykarsk@ump.edu.pl

* Correspondence: jpiontek@ump.edu.pl; Tel.: +48-61-641-83-95.

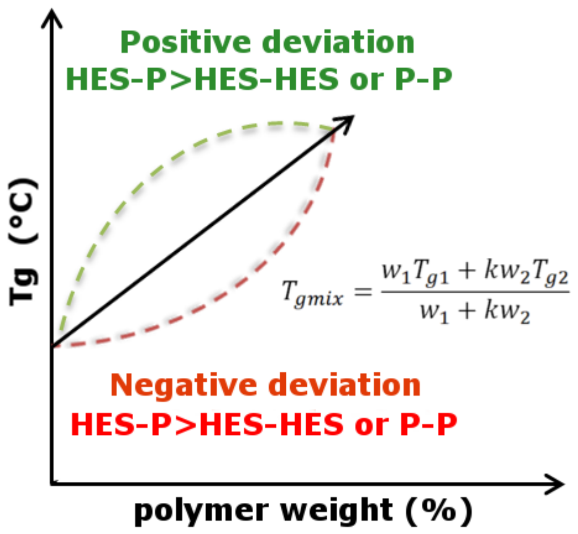

Figure S1 Deviations from the Gordon-Taylor equation; Hes represents hesperidin and P represents carrier. Adapted from reference [1].

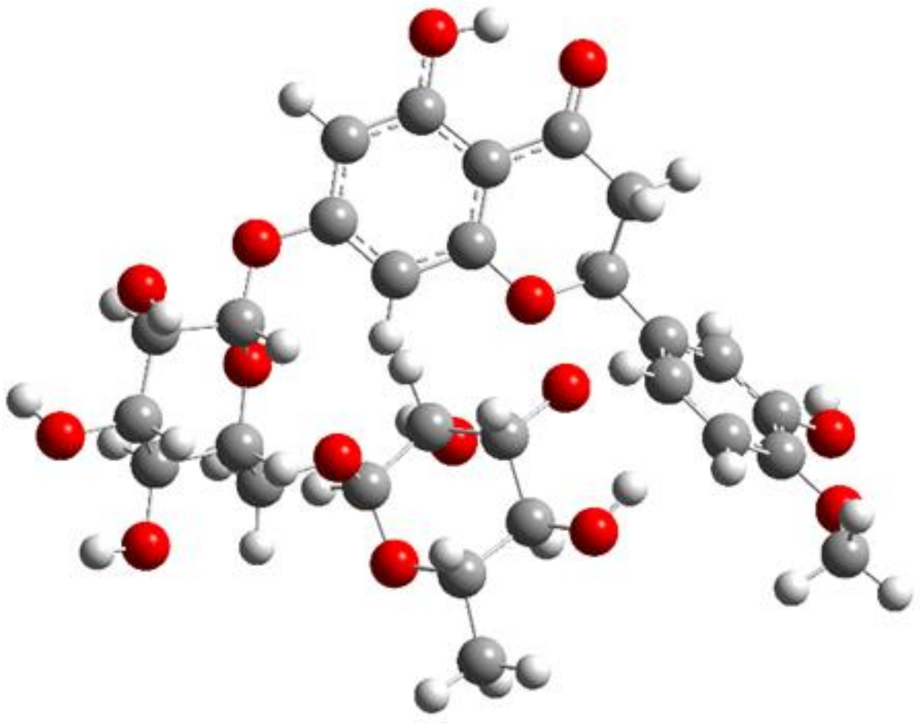

Figure S2. Optimized geometry of the hesperidin.

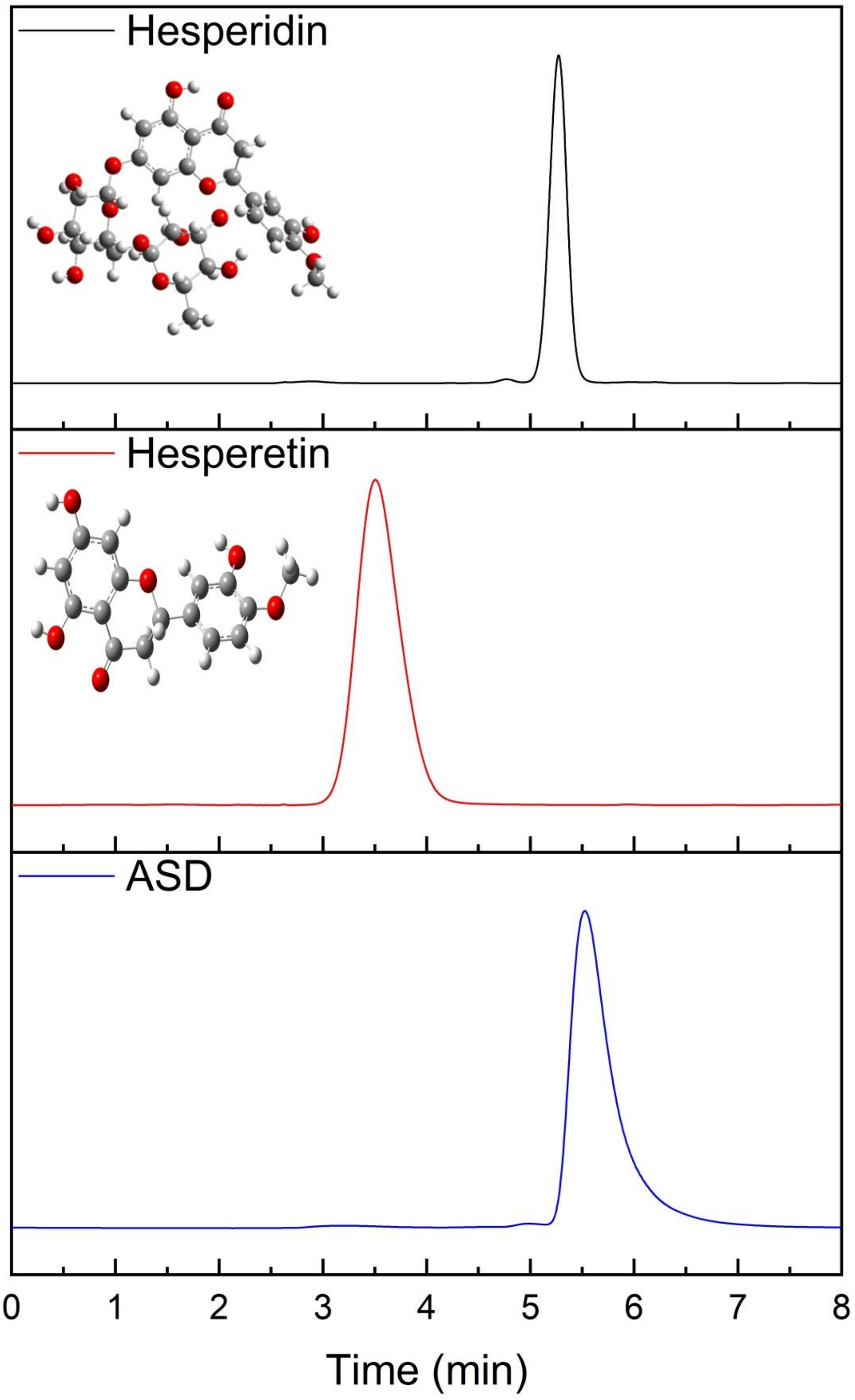

Figure S3. HPLC chromatogram of hesperidin, hesperetin, and ASD of hesperidin.

Stability studies: The Hes-carrier systems were added to 5 mL vials, next they placed in the desiccators containing saturated solutions of sodium chloride (RH = 76.4 %) and stored at temperature T_1=25°C and T_2=60°C [2].

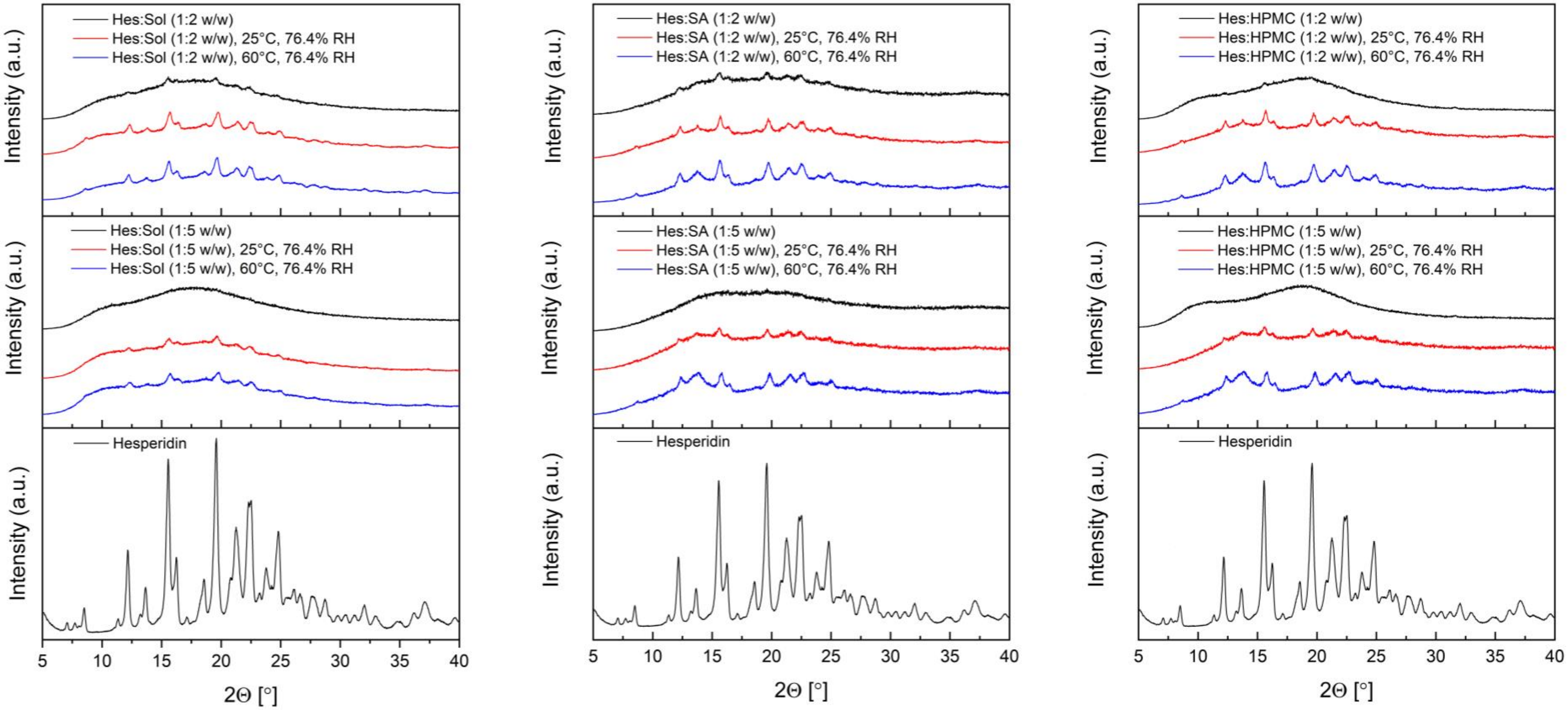

(a) (b) (c)

Figure S4. XRPD analysis: the results of physical stability studies. Hesperidin (Hes), Hes:Sol (1:2 *w/w*), Hes:Sol (1:5 *w/w*) (a); Hes, Hes:SA (1:2 *w/w*), Hes:SA (1:5 *w/w*) (b); Hes, Hes:HPMC (1:2 *w/w*), Hes:HPMC (1:5 *w/w*) (c). The physical stability of hesperidin-carrier systems were evaluated at different conditions 25°/RH = 76.4% and 60°/RH = 76.4% for seven days.

`

Table S1 Selected characteristic bonds (in cm^{-1}) of crystalline hesperidin and amorphous hesperidin. Assignments of hesperidin bands were made based on DFT calculations. Legend: r–rocking, s–stretching, sc–scissoring, t–twisting, w–wagging, def.–deformation

Hesperidin crystalline form	Hesperidin amorphous form	Band assignment
613, 631, 658	635	Def. all molecule
768	762	Breathing B ring
816	804	C–H w at A ring
849		C–H w at A and B rings
876	868	O–C–C s in C ring + C–H r in methylene group at C ring
910	922	C–O–C s in glucose ring + C–H r at CH2 in rutinose
982	984	C–C s in glucose ring + C–O s in glucose and rhamnose ring and between glucose and rhamnose rings + C–H r and O–H r at glucose and rhamnose ring
1022	1018	C–H r at rhamnose ring + C–O s in C–O–C between glucose ring and rhamnose ring
1049	1057	O–H r + C–O s at glucose ring
1094		C–H t in methyl group + C–O r and C–O s at rhamnose ring
1130, 1155	1128, 1157	O–H r and C–H w in methyl group at rhamnose ring
1182		O–H r and C–H r at B ring + C–H r and C–H t at C ring
1206		C–O s between ring A nad glucose ring + C–H r in all molecule
1242		C–O–C assymetric s in ring C + C–H r and O–H r at A ring
1277	1271	C–O–C s + O–H r at B ring + C–H r at C ring
1298	1300	C–O s in C–O–C between A ring and glucose ring + O–H r at A ring + C–H r at C ring and glucose ring
1341	1339	C–C–C s in A ring + C–H r in all molecule
1441	1443	C–H w in methoxy group at B ring
1506		C–H sc in methyl group at B ring
1518	1514	C–C–C s in A ring + O–H r at A ring + C–H r at C ring
1605	1593	O–H r at ring A + C=C s in ring A + C=O s at ring C
1645	1634	O–H r at A ring + C=C s in A ring + C=O s at C ring
2918	2914	C–H s in methoxy group at B ring
2982		C–H s at rhamnose ring
3414		O–H s at A ring
3476		O–H s at rhamnose ring
3543		O–H s at rhamnose ring

`

Table S2. Selected characteristic bonds (in cm⁻¹) of amorphous Hes, Sol, system of Hes:Sol (ratio 1:2, physical mixture), system of Hes:Sol (ratio 1:2), system of Hes:Sol (ratio 1:5, physical mixture), system of Hes:Sol (ratio 1:5). Assignments of hesperidin bands made based on DFT calculations, and carrier based on literature. Legend: r - rocking, s - stretching, sc - scissoring, t - twisting, w - wagging, def. - deformation, * - no information in the literature

Hesperidin amorphous form	Sol	Hes:Sol 1:2	Hes:Sol 1:5	Band assignment
635				Def. all molecule
762		766	766	Breathing B ring
804		808	808	C–H w at A ring
	841	841	841	*
868				O–C–C s in C ring + C–H r in methylene group at C ring
922				C–O–C s in glucose ring + C–H r at CH2 in rutinose
	972	974	974	*
984				C–C s in glucose ring + C–O s in glucose and rhamnose ring and between glucose and rhamnose rings + C–H r and O–H r at glucose and rhamnose ring
1018				C–H r at rhamnose ring + C–O s in C–O–C between glucose ring and rhamnose ring
	1022	1022	1022	*
1057				O–H r + C–O s at glucose ring
	1109			C–O s for ester [3]
1128, 1157		1132		O–H r and C–H w in methyl group at rhamnose ring
	1196	1196	1196	*
	1234	1236	1236	C-O-C s in the ether groups [4]
1271				C–O–C s + O–H r at B ring + C–H r at C ring
1300		1298	1302	C–O s in C–O–C between A ring and glucose ring + O–H r at A ring + C–H r at C ring and glucose ring
	1333	1335	1335	*
1339				C–C–C s in A ring + C–H r in all molecule
	1350	1354	1354	*
	1371	1371	1371	O(C)O or NH [5]
	1422	1423	1423	*
	1439	1443	1443	*
1443				C–H w in methoxy group at B ring

`

Table S2. continuation

Hesperidin amorphous form	Sol	Hes:Sol 1:2	Hes:Sol 1:5	Band assignment
	1476	1479	1479	C–O–C s in the ether groups [6]
1514		1514	1514	C–C–C s in A ring + O–H r at A ring + C–H r at C ring
1593				O–H r at ring A + C=C s in ring A + C=O s at ring C
	1632	1632	1632	C=O s in tertiary amide [6,7] in the caprolactam [4] or C(O)N [5]
1634				O–H r at A ring + C=C s in A ring + C=O s at C ring
	1732	1732	1732	C=O s in the ester [6,7] or OC(O)CH3 [5]
	2859	2859	2859	C–H s
	2926	2926	2926	aliphatic-CH s [7]
2841				C–H s
2914				C–H s in methoxy group at B ring
2934				C–H s
3383		3425	3425	O–H s

Table S3. Selected characteristic bonds (in cm^{-1}) of amorphous Hes, SA, system of Hes:SA (ratio 1:2, physical mixture), system of Hes:SA (ratio 1:2), system of Hes:SA (ratio 1:5, physical mixture), system of Hes:SA (ratio 1:5). Assignments of hesperidin bands made based on DFT calculations, and carrier based on literature. Legend: r - rocking, s - stretching, sc - scissoring, t - twisting, w - wagging, def. - deformation, + - a band is observed in this range

Hesperidin amorphous form	SA	Hes:SA 1:2	Hes:SA 1:5	Band assignment
635				Def.all molecule
762				Breathing B ring
804		+	+	C–H w at A ring
	816	814	814	characteristic of mannuronic acid residues [8]
868				O–C–C s in C ring + C–H r in methylene group at C ring
	881		+	C–H def. of β-mannuronic acid residues [8]
922				C–O–C s in glucose ring + C–H r at CH2 in rutinose
	945			C–O s of uronic acid residues [8]
984				C–C s in glucose ring + C–O s in glucose and rhamnose ring and between glucose and rhamnose rings + C–H r and O–H r at glucose and rhamnose ring
1018		+	+	C–H r at rhamnose ring + C–O s in C–O–C between glucose ring and rhamnose ring
	1026		1026	–OH b [9]
1057				O–H r + C–O s at glucose ring
	1128	1130	1130	C–O s of pyranose ring [8]
1128		1130	1130	O–H r and C–H w in methyl group at rhamnose ring
1271				C–O–C s + O–H r at B ring + C–H r at C ring
1300				C–O s in C–O–C between A ring and glucose ring + O–H r at A ring + C–H r at C ring and glucose ring
1339				C–C–C s in A ring + C–H r in all molecule
	1406		1406	C–OH def. [8]
1443				C–H w in methoxy group at B ring
1514				C–C–C s in A ring + O–H r at A ring + C–H r at C ring
	1599		1599	asymmetric s of carboxylate O–C–O [9,10]
1593				O–H r at ring A + C=C s in ring A + C=O s at ring C

Table S3. Continuation

**Hesperidin amorphous form**	**SA**	**Hes:SA 1:2**	**Hes:SA 1:5**	**Band assignment**
1634				O–H r at A ring + C=C s in A ring + C=O s at C ring
2841				C–H s
2914				C–H s in methoxy group at B ring
	2920			C–H s [8]
2934				C–H s
	3366	3366	3366	O–H s [8]
3383		+	+	O–H s

Table S4. Selected characteristic bonds (in cm^{-1}) of amorphous Hes, HPMC, system of Hes:HPMC (ratio 1:2, physical mixture), system of Hes: HPMC (ratio 1:2), system of Hes: HPMC (ratio 1:5, physical mixture), system of Hes: HPMC (ratio 1:5). Assignments of hesperidin bands made based on DFT calculations, and carrier based on literature. Legend: r - rocking, s - stretching, sc - scissoring, t - twisting, w - wagging, def. - deformation, + - a band is observed in this range

Hesperidin amorphous form	HPMC	Hes:HPMC 1:2	Hes:HPMC 1:5	Band assignment
635				Def. all molecule
762		765	765	Breathing B ring
806		809	809	C–H w at A ring
868				O–C–C s in C ring + C–H r in methylene group at C ring
922				C–O–C s in glucose ring + C–H r at CH2 in rutinose
	945	949	949	C–O–C s in cellulose ether [11,12]
984				C–C s in glucose ring + C–O s in glucose and rhamnose ring and between glucose and rhamnose rings + C–H r and O–H r at glucose and rhamnose ring
1018				C–H r at rhamnose ring + C–O s in C–O–C between glucose ring and rhamnose ring
	1053	1049	1049	C=O s in pyranose ring [12,13]
	1113			C=O bonds in the secondary alcohols [14]
1128		1132		O–H r and C–H w in methyl group at rhamnose ring
	1150			C–O–C s of ether group [15]
	1196	1200	1202	bond of methoxy group [16]
1271		1274	1274	C–O–C s + O–H r at B ring + C–H r at C ring
1300				C–O s in C–O–C between A ring and glucose ring + O–H r at A ring + C–H r at C ring and glucose ring
1339				C–C–C s in A ring + C–H r in all molecule
	1373	1373	1373	C–H b from methyl group [14]
1443		1447	1447	C–H w in methoxy group at B ring
	1454	1445	1449	C–H s from methyl group [14]
1514		1514	1514	C–C–C s in A ring + O–H r at A ring + C–H r at C ring
1593				O–H r at ring A + C=C s in ring A + C=O s at ring C
	1638			axial def. of carbonyl groups in the glucose unit of cellulose [11]

`

Table S4. Continuation

Hesperidin amorphous form	HPMC	Hes:HPMC 1:2	Hes:HPMC 1:5	Band assignment
1634		1637	1637	O–H r at A ring + C=C s in A ring + C=O s at C ring
	1740			C=O bond [17]
	2835	2839	2837	axial def. of the C–H bonds in aliphatic chains [11]
	2905	2897		axial def. of the C–H bonds in aliphatic chains [11]
2914				C–H s in methoxy group at B ring
	2978	2978	2978	axial def. of the C–H bonds in aliphatic chains [11]
2914				C–H s in methoxy group at B ring
2934		2934	2934	C–H s
	3460	3443	3443	O–H s [18]
3383		+	+	O–H s

Table S5. DSC analysis - heating modes of hesperidin

Hesperidin							
**Initial**	**Step 1**	**Step 2**	**Step 3**	**Step 4**	**Step 5**	**Step 6**	**Step 7**
(°C)							
25	↑100	→ 100	↑255	→ 255	↓–25	→ –25	↑277
	10 K min^{-1}	10 min	10 K min^{-1}	5 min	40 K min^{-1}	2 min	40 K min^{-1}

Table S6. DSC analysis - heating modes of Soluplus®, Hes:Sol 1:2, Hes:Sol 1:5

Soluplus®, Hes:Sol 1:2, Hes:Sol 1:5							
**Initial**	**Step 1**	**Step 2**	**Step 3**	**Step 4**	**Step 5**	**Step 6**	**Step 7**
(°C)							
25	↑100	→ 100	↑215	→ 215	↓–25	→ –25	↑215
	10 K min^{-1}	10 min	40 K min^{-1}	5 min	40 K min^{-1}	2 min	40 K min^{-1}

Table S7. DSC analysis - heating modes of alginate sodium, Hes:SA 1:2, Hes:SA 1:5; * - no glass transition was observed

Sodium alginate*, Hes:SA 1:2*, Hes:SA 1:5*							
**Initial**	**Step 1**	**Step 2**	**Step 3**	**Step 4**	**Step 5**	**Step 6**	**Step 7**
(°C)							
25	↑150	→ 150	↓25	→ 25	↑190		
	10 K min^{-1}	10 min	40 K min^{-1}	2 min	40 K min^{-1}		

Table S8. DSC analysis - heating modes of HPMC, Hes:HPMC 1:2, Hes:HPMC 1:5

HPMC, Hes:HPMC 1:2, Hes:HPMC 1:5							
**Initial**	**Step 1**	**Step 2**	**Step 3**	**Step 4**	**Step 5**	**Step 6**	**Step 7**
(°C)							
25	↑100	→ 100	↑215	→ 215	↓25	→ 25	↑215
	10 K min^{-1}	10 min	40 K min^{-1}	5 min	40 K min^{-1}	2 min	40 K min^{-1}

References

1. Kaushal, A.M.; Gupta, P.; Bansal, A.K. Amorphous drug delivery systems: molecular aspects, design, and performance. *Crit. Rev. Ther. Drug Carr. Syst.* **2004**, *21*.

2. Talaczyńska, A.; Lewandowska, K.; Garbacki, P.; Zalewski, P.; Skibiński, R.; Miklaszewski, A.; Mizera, M.; Cielecka-Piontek, J. Solid-state stability studies of crystal form of tebipenem. *Drug Dev. Ind. Pharm.* **2016**, *42*, 238–244, doi:10.3109/03639045.2015.1044902.

3. Thakral, N.K.; Ray, A.R.; Bar-Shalom, D.; Eriksson, A.H.; Majumdar, D.K. Soluplus-Solubilized Citrated Camptothecin—A Potential Drug Delivery Strategy in Colon Cancer. *AAPS PharmSciTech* **2012**, *13*, 59–66, doi:10.1208/s12249-011-9720-0.

4. Chen, Y.; Huang, W.; Chen, J.; Wang, H.; Zhang, S.; Xiong, S. The Synergetic Effects of Nonpolar and Polar Protic Solvents on the Properties of Felodipine and Soluplus in Solutions, Casting Films, and Spray-Dried Solid Dispersions. *J. Pharm. Sci.* **2018**, *107*, 1615–1623, doi:10.1016/j.xphs.2018.02.006.

5. Lan, Y.; Ali, S.; Langley, N. Characterization of Soluplus by FTIR and Raman Spectroscopy. *BASF Corp. Pharma Ingredients Serv. Tarrytown, NY, USA* **2010**, doi:10.13140/2.1.3771.6805.

6. Altamimi, M.A.; Neau, S.H. Investigation of the in vitro performance difference of drug-Soluplus® and drug-PEG 6000 dispersions when prepared using spray drying or lyophilization. *Saudi Pharm. J.* **2017**, *25*, 419–439, doi:10.1016/j.jsps.2016.09.013.

7. Patnaik, S.; Aditha, S.K.; Rattan, T.; Kamisetti, V. Aceclofenac-Soluplus&reg Nanocomposites for Increased Bioavailability. *Soft Nanosci. Lett.* **2015**, *05*, 13–20, doi:10.4236/snl.2015.52003.

8. Leal, D.; Matsuhiro, B.; Rossi, M.; Caruso, F. FT-IR spectra of alginic acid block fractions in three species of brown seaweeds. *Carbohydr. Res.* **2008**, *343*, 308–316, doi:10.1016/j.carres.2007.10.016.

9. Jana, S.; Kumar Trivedi, M.; Tallapragada, R.M. Characterization of Physicochemical and Thermal Properties of Chitosan and Sodium Alginate after Biofield Treatment. *Pharm. Anal. Acta* **2015**, *6*, doi:10.4172/2153-2435.1000430.

10. Leal, D.; Matsuhiro, B.; Rossi, M.; Caruso, F. FT-IR spectra of alginic acid block fractions in three species of brown seaweeds. *Carbohydr. Res.* **2008**, *343*, 308–316, doi:10.1016/j.carres.2007.10.016.

11. Otoni, C.G.; Lorevice, M. V; Moura, M.R. de; Mattoso, L.H.C. On the effects of hydroxyl substitution degree and molecular weight on mechanical and water barrier properties of hydroxypropyl methylcellulose films. *Carbohydr. Polym.* **2018**, *185*, 105–111, doi:10.1016/j.carbpol.2018.01.016.

12. Ali, L.; Ahmad, M.; Usman, M. Evaluation of cross-linked hydroxypropyl methylcellulose graft-methacrylic acid copolymer as extended release oral drug carrier. *Cell. Chem. Technol* **2015**, *49*, 143–151.

13. Hay, W.T.; Fanta, G.F.; Peterson, S.C.; Thomas, A.J.; Utt, K.D.; Walsh, K.A.; Boddu, V.M.; Selling, G.W. Improved hydroxypropyl methylcellulose (HPMC) films through incorporation of amylose-sodium palmitate inclusion complexes. *Carbohydr. Polym.* **2018**, *188*, 76–84, doi:10.1016/j.carbpol.2018.01.088.

`

14. Akinosho, H.; Hawkins, S.; Wicker, L. Hydroxypropyl methylcellulose substituent analysis and rheological properties. *Carbohydr. Polym.* **2013**, *98*, 276–281, doi:10.1016/j.carbpol.2013.05.081.

15. Langkilde, F.W.; Svantesson, A. Identification of celluloses with Fourier-Transform (FT) mid-infrared, FT-Raman and near-infrared spectrometry. *J. Pharm. Biomed. Anal.* **1995**, *13*, 409–414, doi:10.1016/0731-7085(95)01298-Y.

16. Gustafsson, C.; Nyström, C.; Lennholm, H.; Bonferoni, M.C.; Caramella, C.M. Characteristics of Hydroxypropyl Methylcellulose Influencing Compactibility and Prediction of Particle and Tablet Properties by Infrared Spectroscopy. *J. Pharm. Sci.* **2003**, *92*, 494–504, doi:10.1002/jps.10323.

17. Shi, S.-C.; Wu, J.-Y.; Huang, T.-F. Raman, FTIR, and XRD study of MoS2 enhanced hydroxypropyl methylcellulose green lubricant. *Opt. Quantum Electron.* **2016**, *48*, 474, doi:10.1007/s11082-016-0748-y.

18. Huang, H.-C.; Chen, L.-C.; Lin, S.-B.; Chen, H.-H. Nano-biomaterials application: In situ modification of bacterial cellulose structure by adding HPMC during fermentation. *Carbohydr. Polym.* **2011**, *83*, 979–987, doi:10.1016/j.carbpol.2010.09.011.
